# A comparative study on the traditional Indian Shodhana and Chinese processing methods for aconite roots by characterization and determination of the major components

**DOI:** 10.1186/1752-153X-7-169

**Published:** 2013-10-25

**Authors:** Yogini Jaiswal, Zhitao Liang, Peng Yong, Hubiao Chen, Zhongzhen Zhao

**Affiliations:** 1 School of Chinese Medicine, Hong Kong Baptist University, Kowloon, Hong Kong Special Administrative Region, P. R. China; 2 Institute of Medicinal Plant Development, Peking Union Medical College and Chinese Academy of Medical Sciences, Haidian District, Beijing, People’s Republic of China

**Keywords:** *Aconitum heterophyllum* wall, *A*. *carmichaelii* Debx, *A*. *kusnezoffii* Reichb, Ranunculaceae, Detoxification, Ayurveda, Traditional Chinese medicine, UHPLC-Q-TOF-MS, Diester diterpenoid alkaloids

## Abstract

**Background:**

Aconitum is an indispensable entity of the traditional medicine therapy in Ayurveda and Traditional Chinese medicine (TCM), in spite of its known fatal toxicity characteristics. The prolonged use of this drug, irrespective of its known lethal effects, is governed by the practice of effective detoxification processes that have been used for decades. However, the processing methods of Ayurveda and TCM are different, and no comparative study has been carried out to evaluate their differences.

The objective of the present study was to carry out comparative chemical profiling of the roots of *Aconitum heterophyllum* Wall, *A. carmichaelii* Debx., and *A. kusnezoffii* Reichb. after application of two detoxification methods used in Ayurveda and one method used in TCM .

**Results:**

Analysis of the processed samples was carried out by ultra-high performance liquid chromatography combined with quadrupole time-of-flight mass spectrometry (UHPLC-QTOF/MS). The results obtained in the study demonstrate that all three processing methods used in Ayurveda and TCM effectively extract the diester diterpenoid alkaloids and led to their conversion into monoester diterpenoid alkaloids. The efficiency of the processes in reduction of toxic alkaloid contents can be stated as: Processing with water > Shodhana with cow milk > Shodhana with cow urine. The analysis method was validated as per ICH-Q2R1 guidelines and all the parameters were found to comply with the recommendations stated in the guidelines.

**Conclusions:**

There have been no reports till date, to compare the processing methods used in Ayurveda with the methods used in TCM for detoxification of aconite roots. Our study demonstrates that, these methods used in both the traditional systems of medicine, efficiently detoxify the aconite roots. Amongst the three selected procedures, the TCM method of decoction with water is the most efficient. Through experimental evidences, we prove the conversion of toxic diester diterpenoid alkaloids to relatively safer monoester diterpenoid alkaloids. Thus, this study demonstrates that comparative study on the traditional experiences accumulated in different medical systems is useful for expanding their respective applications.

## Background

For centuries, Aconite has been highly regarded in the traditional medicine of China (TCM) and of India (Ayurveda) [1,2]. The genus *Aconitum* (family Ranunculaceae) has more than 300 species worldwide, of which more than 166 are found in China and India [3,4]. The roots of *Aconitum heterophyllum* Wall (Atis) are used as medicine in India, and its preparations are mentioned in the Ayurvedic Pharmacopoeia and Ayurvedic formulary of India [5,6]. The processed roots of *A*. *carmichaelii* Debx. (Zhichuanwu) and *A*. *kusnezoffii* (Zhicaowu) have been widely used in China and are listed in the Chinese Pharmacopoeia [7]. These plants are used for treating rheumatalgia, rheumatic arthritis, cold, pain and other ailments [6-11].

As is well known, the unprocessed Aconite root, if ingested, causes fatal toxicities [9-11]. In India and China, many medicinal herbs are subjected to specific treatments before they are used as materia medica. The history of the alchemy of aconite processing in India dates back to the 5th and 6th centuries, and received wide acceptance during the 8th and 9th centuries A.D. After the 8th century the ancient science of pharmacy called “*Rasashastra”* was used routinely by herbal medical practitioners. Processing methods in Ayurveda (called *Samskaras*) consist of two stages: The *Shodhana* (purification or detoxification) and *Bhaishajya kalpana* (formulation methods). The process of *Shodhana* involves treatment of the drug with “Goumutra” (cow urine) and cow milk [12-16]. About 200 medical texts that describe the *Shodhana* process, have been written in various languages. Amongst these 200 published texts, “*Charaka Samhita*” is said to establish the basic concepts for processing herbal medicines [12-15].

In the ancient TCM records, various methods for detoxification of Aconite roots and 600 different formulations prepared from processed aconite are documented [17]. The use of the processed form of aconitum was first documented in the ‘Shennong Materia Medica’ (Sheng-nong Ben Cao Jing) written in the Eastern Han Dynasty (24–220 AD); and this herb is an integral part of traditional medicine practices for treatment of arthralgia, colds, cardiac problems, diarrhoea, and oedema [18]. Two classic ancient monographs, namely Lei Gong Processing Handbook (Lei Gong Pao Zhi Lun, written in 500 AD) and Processing Methodology (Pao Zhi Da Fa, published in 1662), have mentioned the processing method of Aconite roots [19,20]. According to the Chinese pharmacopoeia (2010 edition), various procedures involving treatment with mineral salt water and decoction with water were recorded for processing the roots of *A*. *carmichaelii* and *A*. *kusnezoffii* to produce the medicinal products called “Fuzi” and “Zhicaowu”, respectively [7,17]. Among those processing methods, boiling with water for processing the roots of *Aconite* is the simplest methods and is selected for the present comparative study.

**Figure 1:**
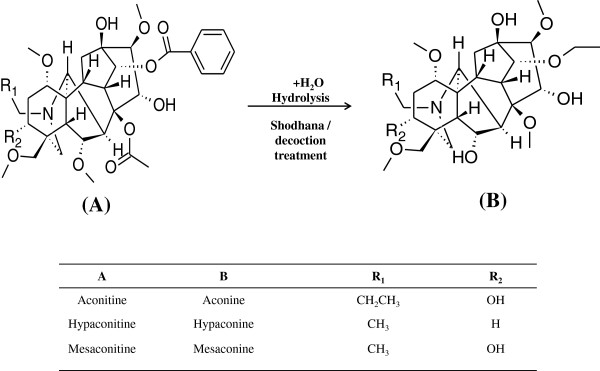
Structural changes affected by hydrolysis leading to detoxification of toxic aconitum alkaloids.

The specific treatments claim to enhance the efficacy and reduce the toxicity of crude drugs by alteration of their pharmacodynamic properties. For Aconite roots, the pharmacodynamic and toxicity level changes occur due to modifications in the structures of Diester Diterpenoid Alkaloids (DDA’s) after processing, leading to formation of monoester diterpenoid alkaloids (MDA’s) [21-23]. The reaction hypothesised for these changes in the chemistry of Diester Diterpenoid Alkaloids is indicated in Figure 1[17,24]. There have been several pharmacological studies that demonstrate the reduction in the toxicity of the processed aconitum due to *Shodhana* treatment [22,25]. Although this process has been used for more than 200 years, the alchemy behind these processing strategies still lacks evidence-based scientific validation and needs further understanding. Moreover, there have been no chemical profiling studies that can substantiate and verify the chemical transformations resulting in radical reduction in the toxicity of Aconitum due to *Shodhana*.

The *Shodhana* process is not particularly mentioned only for processing of *Aconitum heterophyllum* in the Ayurvedic Pharmacopoeia of India. There have been no reports published for the quantitation of toxic alkaloids of these three species of Aconitum processed by *Shodhana* method and for the comparative study of the two traditional processing methods. In contrast, the TCM processes have been explored extensively through pharmacological and analytical studies [8,24-33]. Toxicity of nine types of decoction pieces from the daughter root of *A*. *carmichaelii* (Fuzi) based on chemical analysis of diester diterpenoid alkaloids was assessed in our previous study [28].

The aim of the present study was to carry out a comparative study of these two detoxification methods belonging to two different traditional systems of medicine, with a view of providing insights into the changes in the phytochemical composition of the processed forms. The changes were studied with respect to the most toxic components of Aconite *viz.* aconitine, mesaconitine, and hypaconitine used as markers in the study, as these are the major determinants of aconite toxicity. A ultra-high performance liquid chromatography combined with quadrupole time-of-flight mass spectrometry (UHPLC-Q-TOFMS) method, that has advantages of high sensitivity and mass accuracy over other chromatographic detection methods, was used for the analysis of the samples. The method used was developed and validated as per ICH (Q2-R1) guidelines for validation of analytical methods [34].

## Results and discussion

### Validation of the developed analytical method

**Figure 2:**
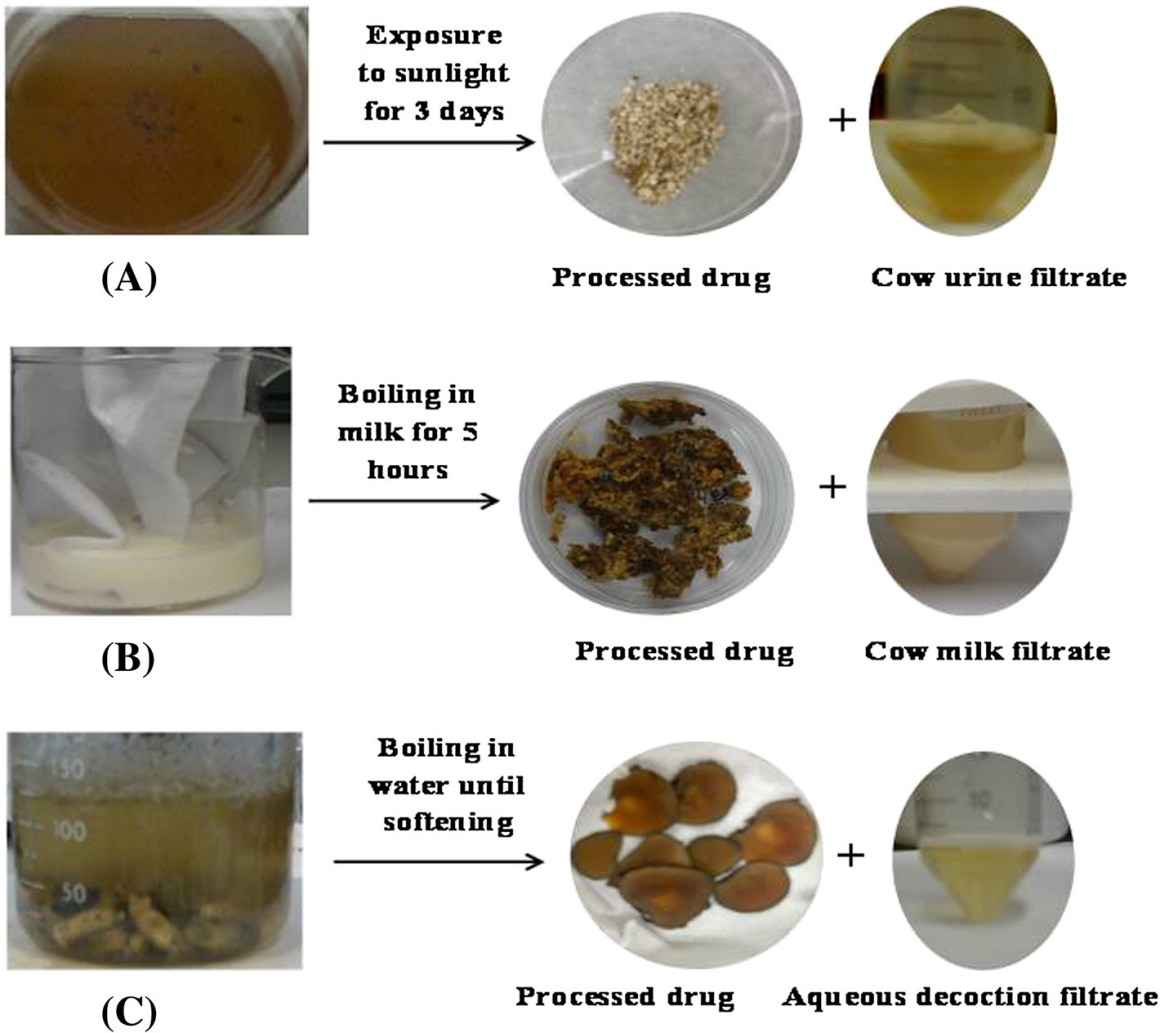
Representation of Shodhana and TCM detoxification process applied for selected Aconitum species (A) Drug treated with cow urine (B) Drug boiled in cow milk (C) Pre-soaked drug subjected to decoction with water.

**Figure 3:**
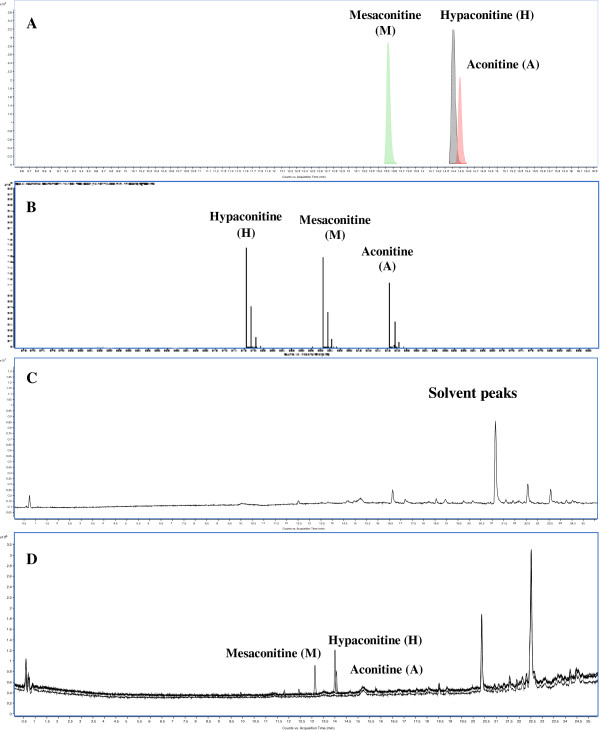
**Representative LC-MS chromatograms of marker compounds and solvent blank used in analysis (A) Extracted Compound Chromatogram of marker compounds (B) MFE spectrum of markers indicating the *m/z *ratios (C) Base peak chromatogram of methanol used as solvent (D) TIC overlay spectra of markers compounds.** Abbreviations used: A- Aconitine, M-Mesaconitine, H-Hypaconitine.

The method developed for analyzing the detoxified samples was validated as per ICH guidelines Q2R1. A pictorial representation of the detoxification strategy used is shown in Figure 2, and the LC-MS spectra of marker compounds are shown in Figure 3.

The linearity ranges for all marker compounds were estimated, and satisfactory regression coefficient (r^2^ ≥ 0.99) values were obtained for all three selected markers. The calibration ranges for aconitine (r^2^ = 0.9988), mesaconitine (r^2^ = 0.9994) and hypaconitine (r^2^ = 0.9990) were between 2.0 – 100 ng mL^-1^. The calibration curve equations obtained for all the three markers are shown in Table 1. The limits of detection (LOD) of aconitine, mesaconitine and hypaconitine were found to be 0.383 ng mL^-1^, 0.438 ng mL^-1^ and 0.088 ng mL^-1^, respectively. The limits of quantitation (LOQ) for aconitine, mesaconitine and hypaconitine were 1.15 ng mL^-1^, 1.31 ng mL^-1^ and 0.264 ng mL^-1^ respectively. All the determinations were carried out with sample concentrations within the calibration range selected for each marker compound. The representative Extracted Compound Chromatogram (ECC) chromatogram, Molecular Feature Extraction (MFE) spectrum, and Total Ion Chromatogram (TIC) overlay spectra of markers along with Base Peak Chromatogram (BPC) of methanol used as solvent are shown in Figure 3.

**Table 1:** Results of linearity studies

**Parameters**	**Aconitine**	**Mesaconitine**	**Hypaconitine**
Calibration curve equation	y = 33693.02x – 625.504	y = 29797.96x – 51913.504	y = 19022.440x – 24159.317
Correlation coefficient value (r^2^)	0.9988	0.9994	0.9990
LOD (ng mL^-1^)	0.383	0.438	0.088
LOQ (ng mL^-1^)	1.15	1.315	0.264

* All the determinations were performed in triplicate at 6 predetermined concentrations.

The precision studies were carried out with three assays, the Interday precision, Intraday precision, and repeatability. The average contents for all markers were calculated for each determination, and the values are indicated in Table 2. All the RSD values are expressed as percentages, and all of them fall within the limits (≤ 5%) as stated by the ICH guidelines. For Interday precision, the % RSD values (n = 3) for aconitine, mesaconitine and hypaconitine were found to be 0.037, 0.064, and 0.107, respectively. For Intraday precision, the% RSD values (n = 5) for aconitine, mesaconitine and hypaconitine were 0.243, 0.249 and 0.477, respectively. The repeatability studies were carried out with 5 replicate determinations of the same samples weighed individually five times. The values of % RSD (n = 5) obtained in repeatability studies for aconitine, mesaconitine and hypaconitine were 0.530, 0.324 and 0.200%, respectively. The results obtained suggest that the developed method is precise enough to measure the contents in close precision for replicate analysis within the same day, for three consecutive days and for multiple measurements of the same sample on the same day using the optimized chromatographic parameters of the method.

**Table 2:** Results of precision studies

**Parameters**	**Aconitine**	**Mesaconitine**	**Hypaconitine**
	**(ng mL**^**-1**^**)**	**(ng mL**^**-1**^**)**	**(ng mL**^**-1**^**)**
**Interday precision**			
Content values*^a^ (*Mean* ± S. D.)	4.372 ± 0.001	4.764 ± 0.003	4.523 ± 0.004
*% RSD*	0.037	0.064	0.107
**Intraday precision**			
Content values*^b^ (*Mean* ± S. D.)	4.160 ± 0.010	4.608 ± 0.011	4.352 ± 0.020
*% RSD*	0.243	0.249	0.477

*^a^values for three replicate determinations; *^b^values for five replicate determinations data were collected.

The recovery study for all the three marker compounds was performed at three levels by adding standard compounds at 50, 100 and 150% of their concentrations in the sample. The results of recovery study are shown in Table 3. Overall, the recoveries were found to be within the range of 85-108% of the expected concentrations of the standards. Results indicate that the method is accurate and precise enough to recover the compounds of interest from a complex herbal sample matrix. Thus, based upon the results obtained for the validation parameters, we suggest that the validated method can be used for the present study.

**Table 3:** Results of recovery studies

**Accuracy**	**Aconitine**	**Mesaconitine**	**Hypaconitine**
	*Mean ± S. D.	*Mean ± S. D.	*Mean ± S. D.
***Low level Spike (50%)***			
Amount of standard added (ng)	2.200	2.460	2.260
Amount of standard recovered (ng)	2.313 ± 0.012	2.316 ± 0.002	2.203 ± 0.004
% Recovery	105.150	94.174	97.516
***Intermediate level Spike (100%)***			
Amount of standard added (ng)	4.440	4.920	4.520
Amount of standard recovered (ng)	4.159 ± 0.007	5.326 ± 0.013	3.928 ± 0.010
% Recovery	93.671	108.250	86.920
***High level Spike (150%)***			
Amount of standard added (ng)	6.640	7.380	6.78
Amount of standard recovered (ng)	6.573 ± 0.005	7.330 ± 0.006	6.474 ± 0.004
% Recovery	98.992	99.322	95.500
Avg. Recovery	99.271%	100.582%	93.312%
RSD %	0.057	0.070	0.060

*(n = 3) for each of the values mentioned for the above determinations.

**Figure 4:**
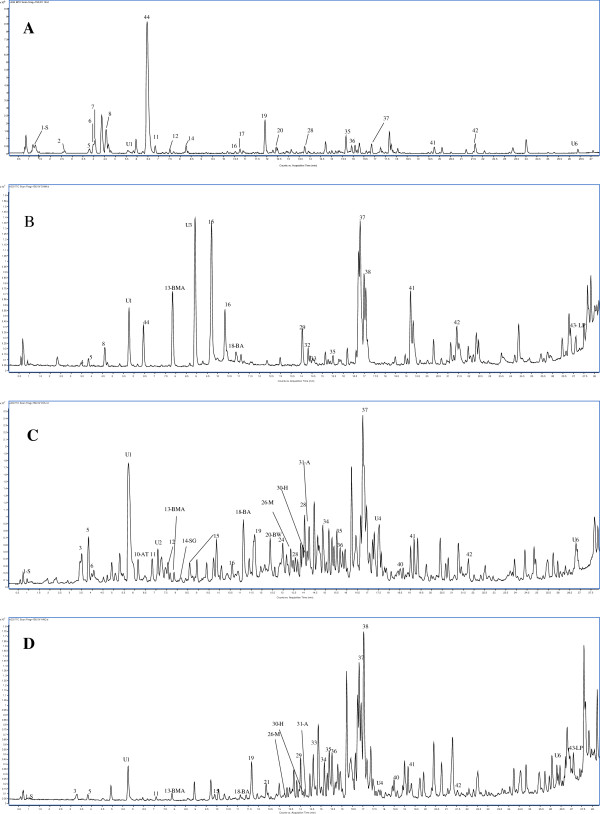
**Representative LC-MS base peak chromatograms of roots of *A. heterophyllum *before and after processing (A) Unprocessed sample (B) Processed with cow milk (C) Processed with cow urine (D) Processed with water.** Abbreviations used: AT- Atisine, BW- Beiwutine, BA- benzoylaconine, A- Aconitine, M-Mesaconitine, H-Hypaconitine, LP- Lipo-14-O-anisoylbikhaconine, S-(−) Salsolinol, SG-Songoramine, BMA- benzoylmesaconine.

**Figure 5:**
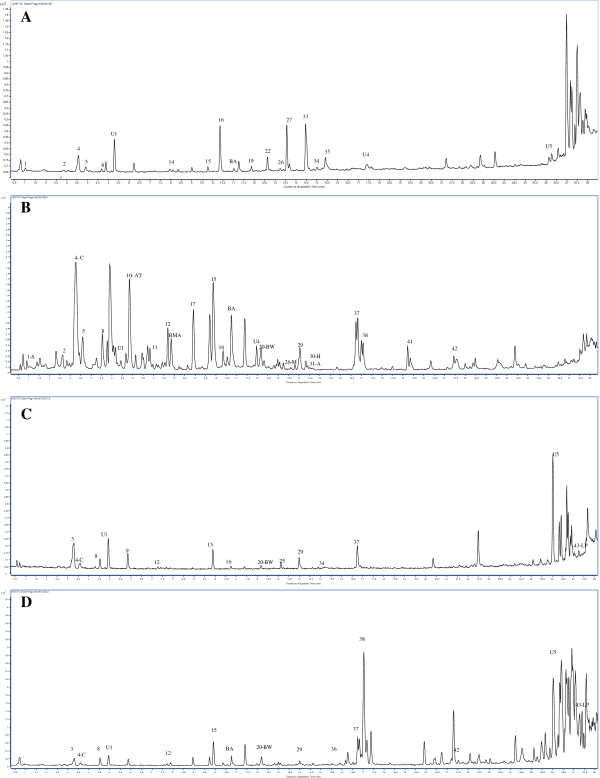
**Representative LC-MS base peak chromatograms of roots of *A*. *carmichaelii *before and after processing (A) Unprocessed sample (B) Processed with cow milk (C) Processed with cow urine (D) Processed with water.** Abbreviations used: AT- Atisine, BMA- Benzoylmesaconine, BA- Benzoylaconine, SB-Senbusine-A- Aconitine, H-Hypaconitine, LP-Lipo-14-O-anisoylbikhaconine, S-(−) Salsolinol, C- carmichaelline, BW-Beiwutine, M-Mesaconitine.

**Figure 6:**
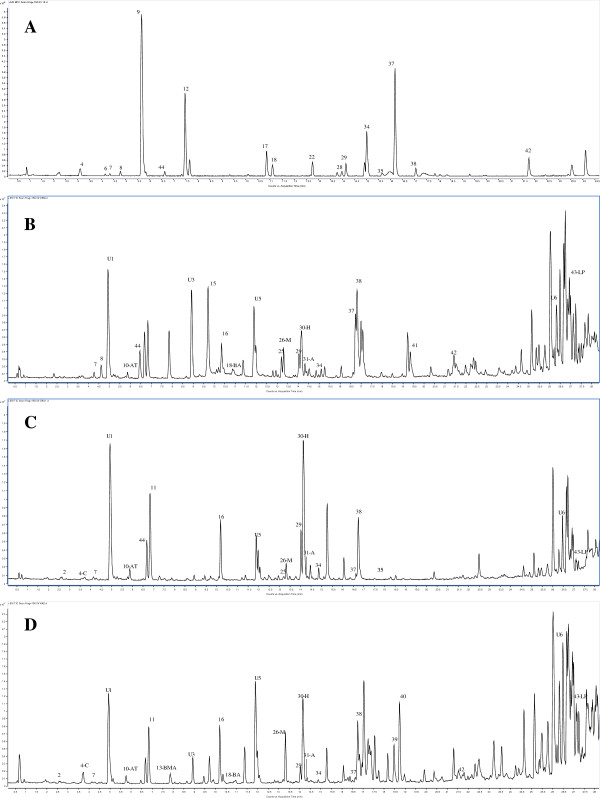
**Representative LC-MS base peak chromatograms of roots of *A*. *kusnezoffii *before and after processing (A) Unprocessed sample (B) Drug boiled in cow milk (C) Processed with cow urine (D) Processed with water**. Abbreviations used: S-(−) Salsolinol, AT- Atisine, SG- Songoramine, BA- Benzoylaconine, A- Aconitine, H-Hypaconitine, M-Mesaconitine, BW- Beiwutine, BMA- Benzoylmesaconine, LP- Lipo-14-O-anisoylbikhaconine.

The representative LC-MS BPC chromatograms of all the samples indicating the profiles of various components are shown in Figures 4, 5, 6 and Additional file 1: Figures S1, Additional file 2: Figure S2, Additional file 3: Figure S3 of the additional data files. Overall 50 constituents in the samples were identified, that included 44 known components and 6 unknown components represented in the additional data files (Additional file 4: Table S1 of additional data file). The marker compounds in samples were estimated by comparing the retention times and mass-to-charge (*m/z*) ratios with the reference standards. Aconitine and mesaconitine exhibited retention times of 14.5 min and 13.5 min with mass-to-charge (*m/z*) ratios of 646.322 and 632.306, respectively. For hypaconitine, the retention time was found to be 14.4 min with a *m/z* ratio of 616.311. The other component peaks were identified based upon the *m/z* values and information available in literature [27,35-37]. To exclude the interferences of the endogenous and exogenous substances that can be present in filtrate of cow urine and cow milk after extraction, the list of components identified in the blank controls with their m/z values, retention times, and masses are mentioned in Additional file 5: Table S2 (additional data file).

Reports in literature suggest that the DDA alkaloids upon hydrolysis form MDA compounds. We found similar results in our previous unpublished studies related to metabolite analysis of various Aconitum species [35-37]. This conversion occurs by loss of groups as BzOH, H_2_O, MeOH, and CO. The loss of these chemical group leads to loss in masses of 122 Da, 18 Da, 32 Da, and 28 Da respectively. The loss of acetic acid group is a characteristic feature for formation of MDA compounds from DDA compounds and results into a loss of 60 Da. Through LC-MS analysis, the occurrence of this reaction and formation of MDA compounds can be evidently traced by detection of components with masses similar to the MDA compounds. The MDA compounds formed by molecular changes in DDA compounds are reported in literature, and their *m/z* values are represented in Additional file 6: Figure S5 (see additional data). In our study we observed the formation of MDA compounds such as benzoyl mesaconine and benzoyl aconine due to hydrolysis reaction during processing. This is evident by the presence of components with masses equal to the reported mass values for these MDA compounds. The LC-MS mass spectra of the filtrates were derived and the formation of hydrolyzed constituents was ascertained. The MDA compounds identified in the processed drugs and the filtrates of mediums used for processing are indicated by suitable abbreviations in LC-MS base peak chromatograms in Figures 4, 5, 6 and ESI scan spectra in Additional file 7: Figure S4, Additional file 6: Figure S5 (additional data) respectively. This provides evidences for the scientific basis of traditional alchemy of detoxification of Aconitum, caused by the hydrolytic conversion of DDA to MDA compounds. To further substantiate the claims of our study, the quantitation of marker alkaloids in the processed and unprocessed drug and the filtrates of various extraction mediums used, was carried out. The estimated contents of the markers in all the samples of each of the three selected species are shown in Table 4. It was observed that, the processing with water extracted the highest amount of marker alkaloids into the solvent filtrate as compared to cow urine and cow milk. The content of aconitine, mesaconitine and hypaconitine in the decoction filtrates was higher than their contents in the processed drug. In the unprocessed drug sample, the content of aconitine, mesaconitine and hypaconitine in *A*. *heterophyllum* was lower as compared to *A*. *kusnezoffii* and *A*. *carmichaelii*.

**Table 4:** Content of toxic alkaloids in various processed and unprocessed samples of Aconitum roots

**Sample**	**Aconitine**	**Mesaconitine**	**Hypaconitine**
	**(mg/kg)**	**(mg/kg)**	**(mg/kg)**
	*Mean ± S. D.	*Mean ± S. D.	*Mean ± S. D.
***A*. *heterophyllum***			
Unprocessed drug material	11.310 ± 0.629	9.680 ± 0.327	13.760 ± 0.022
Drug processed with Cow milk	1.050 ± 0.146	1.964 ± 0.504	0.943 ± 0.016
Filtrate of cow milk decoction	0.274 ± 0.052	1.085 ± 0.62	0.361 ± 0.058
Drug processed with Cow urine	4.700 ± 0.034	2.127 ± 0.433	3.284 ± 0.856
Filtrate of cow urine extraction	5.209 ± 1.426	2.985 ± 0.798	4.076 ± 065
Drug processed with water	1.900 ± 0.002	0.962 ± 0.275	0.119 ± 0.032
Filtrate of aqueous decoction	2.198 ± 0.968	1.086 ± 0.724	0.212 ± 0.105
***A*. *carmichaelii***			
Unprocessed drug material	16.265 ± 0.082	15.305 ± 0.605	16.245 ± 0.538
Drug processed with Cow milk	2.078 ± 0.002	1.047 ± 0.748	1.797 ± 0.049
Filtrate of cow milk decoction	4.284 ± 0.440	1.288 ± 0.249	1.039 ± 0.138
Drug processed with Cow urine	5.536 ± 0.008	3.518 ± 0.322	4.405 ± 0.122
Filtrate of cow urine extraction	1.361 ± 0.688	3.428 ± 0.127	6.177 ± 1.720
Drug processed with water	1.150 ± 0.445	1.729 ± 0.275	2.829 ± 0.007
Filtrate of aqueous decoction	2.615 ± 0.578	2.40 ± 0.775	3.174 ± 0.583
***A*. *kusnezoffii***			
Unprocessed drug material	14.405 ± 0.386	14.150 ± 0.605	18.135 ± 0.229
Drug processed with Cow milk	3.960 ± 0.088	4.389 ± 0.060	1.720 ± 0.069
Filtrate of cow milk decoction	7.817 ± 1.707	8.502 ± 1.71	13.658 ± 4.21
Drug processed with Cow urine	11.125 ± 0.101	4.865 ± 0.220	3.088 ± 0.053
Filtrate of cow urine extraction	2.258 ± 0.231	6.689 ± 0.218	3.840 ± 0.33
Drug processed with water	2.050 ± 0.056	2.448 ± 0.150	1.067 ± 0.070
Filtrate of aqueous decoction	4.947 ± 1.749	4.086 ± 1.095	2.863 ± 0.817

*(n = 3) for each of the values mentioned for the above determinations.

### Comparison of samples treated with cow milk

The LC-MS chromatogram of *A*. *heterophyllum* for unprocessed drug (Figure 4A) compared to milk processed drug (Figure 4B) shows a decrease in the number of toxic components. We observed the formation of lipo-14-O-anisoylbikhaconine, benzoylmesaconine, and benzoylaconine that are distinctively present only in the processed drug sample and not present in the unprocessed drug. Compounds belonging to the less toxic and monoester diterpene alkaloids (MDA) category, *viz.* hokbusine b, bullatine B, talatizamine, ignavine, isodelphinine, and delgrandine, were also observed. The content of aconitine, mesaconitine and hypaconitine in the unprocessed drug calculated in terms of dry powder form was 11.310, 9.680 and 13.760 mg/kg which were found to be reduced to 1.050, 1.964 and 0.943 mg/kg respectively in the processed drug. This indicated that a 10–12 fold decrease in the content of selected DDA contents occurred in the processed sample (see Table 4). The milk filtrate obtained after treatment of the drug, (see Additional file 1: Figure S1-A of additional data files) was found to contain atisine, (−) salsolinol, senbusine, songoramine, senbusine-C and deltaline (the components belonging to the more toxic class of aconitum alkaloids) along with other less toxic compounds.

In the detoxification process of *A*. *carmichaelii* with cow milk*,* the unprocessed drug profile (see Figure 5A) shows presence of lesser number of components as compared to the processed drug (Figure 5B). The content of aconitine, mesaconitine and hypaconitine were found to be reduced from 16.265, 15.305 and 16.245 mg/kg to 2.078, 1.047 and 1.797 mg/kg, respectively. An overall 8–15 fold decrease in the content of the marker alkaloids was observed as shown in Table 4. The milk filtrate was found to contain the toxic components like hypaconitine, aconitine, mesaconitine, beiwutine, atisine, carmichaelline, (−) salsolinol, and songoramine, as seen in Additional file 1: Figure S1-B of the additional data file. The formation of unknown compounds U1 and U5 along with lipo-14-O-anisoylbikhaconine, benzoylaconine, beiwutine and benzoylmesaconine was also evident in processed *Aconitum carmichaelii* (see Figure 5B).

In the case of *A*. *kusnezoffii,* the unprocessed drug was found to contain fewer components as compared to the processed samples (see Figure 6A and B). This indicates the possible formation of aconines and benzoylaconine products along with other non-toxic components due to hydrolysis. As observed in Additional file 1: Figure S1-C of the additional data file, the milk extract of *Aconitum kusnezoffii* contained (−) salosinol, mesaconitine, aconitine, hypaconitine, beiwutine and the unknown compound U5 in the unprocessed drug. In the processed drug (Figure 6B), benzoyl aconine, 14-O-veratoylneoline, isodelphinine, lipo-14-O-anisoylbikhaconine, aconifine, lycoctonine and bullatine B were found. The contents of aconitine, mesaconitine and hypaconitine decreased from 14.405, 14.150 and 18.135 mg/kg to 3.960, 4.389 and 1.720 mg/kg, respectively, indicating an overall 4–8 fold decrease in the content of the alkaloidal components (see Table 4).

There are several reports which suggest that alkaloidal components are easily extracted into milk [38-40], and they are unstable at elevated temperatures [17]. Also, the pH of milk (ca. pH 6.5) that is acidic compared to plasma, leads to the transfer of basic compounds like plant alkaloids into the milk [41]. We suggest that affinity of alkaloids towards milk due to differences in the pH and their instability at elevated temperatures are the factors that support this ancient alchemical process and our experimental hypothesis for the use of cow milk in detoxification of aconitum roots.

### Comparison of samples treated with cow urine

In the detoxification process with cow urine, it was observed that the unprocessed drug of *A*. *heterophyllum* (see Figure 4A) has comparatively fewer peaks than the processed drug (Figure 4C) and the urine filtrate (Additional file 2: Figure S2-A of the additional data file). Although not as efficient as milk, cow urine could also essentially extract toxic components like (−) salsolinol, atisine, deltaline, mesaconitine, aconitine, songoramine, and benzoylaconine (as seen in Additional file 2: Figure S2-A of the additional data file). As shown in Figure 4C, processed *A*. *heterophyllum* contains the compounds benzoylmesaconine, anthranoyllcoctonine, lipo-14-O-anisoylbikhaconine and neojiangyouaconitine. The content of markers in the processed drug showed a 3–4 fold decrease from 11.310, 9.680 and 13.760 mg/kg to 4.700, 2.127 and 3.284 mg/kg for aconitine, mesaconitine and hypaconitine compared to the unprocessed drug (Table 4).

Compared with the LC-MS BPC profile of unprocessed *A*. *carmichaelii* (Figure 5A), the profile of urine filtrate and the processed drug, both showed a marked increase in number of components (Additional file 2: Figure S2-B and Figure 5C). The urine filtrate was found to contain benzoylaconine, songorine, mesaconitine, beiwutine, hypaconitine, aconitine, 3-deoxyaconitine and carmichaelline. The processed drug was found to contain lipo-14-O-anisoylbikhaconine, aconifine, delbrusine, 14-O-anisoylneoline, and lycoctonine. A 3–4 fold reduction was observed in the initial concentration of the toxic components compared with the unprocessed drug (see Table 4). The contents of aconitine, mesaconitine and hypaconitine were found to decrease to 5.536, 3.518 and 4.405 mg/kg, respectively.

In *A*. *kusnezoffii,* the unprocessed drug (Figure 6A) contains fewer components as compared to the urine filtrate (Additional file 2: Figure S2-C of additional data file) and the processed drug (Figure 6C). The cow urine filtrate obtained after processing, shows the presence of benzoylaconine, hypaconitine, aconitine, beiwutine, and mesaconitine. The processed drug shows difference in the presence of new components such as lipo-14-O-anisoylbikhaconine, senbusine-C, and delphatine. The LC-MS profile observations showed a 4–6 fold decrease in the content of toxic alkaloids, with decreased values of aconitine, mesaconitine and hypaconitine as 11.125, 4.8653 and 3.088 mg/kg, respectively (see Table 4).

The ancient literature and modern scientific findings suggest that cow urine can enhance the potency and bioavailability of drugs, reduce toxicity and potentiate the efficacy of drugs without itself acting as a drug [42-52]. In our experiments, the cow urine used for processing was found to have pH 6.9. The acidic pH of the urine and exposure to heat through sunlight may be the possible reasons for the detoxification reaction of aconitum alkaloids.

### Comparison of samples treated by aqueous decoction

The Chinese pharmacopeia states several methods for processing of aconite roots by treatment with mineral salt, steaming and decoction with water [7]. We selected the process of boiling with water for our study because water is the most easily and widely available solvent and because it is commonly used for detoxification of several other drugs. The TCM method was found to be the most effective method for detoxification as inferred from the overall decrease observed in the toxic alkaloid marker contents in comparison to the values obtained for the other two methods of *Shodhana* (see Table 4). For *A*. *heterophyllum* unprocessed drug, the results (see Figure 4A) clearly show that there is an effective extraction of components in the aqueous filtrate. This may be due to some hydrolytic reaction (Figure 4D). The components extracted in the aqueous filtrate are greater in number and more toxic in nature than the components present in the processed drug (Additional file 3: Figure S3-A of additional data file). Most of the components found in the aqueous filtrate, viz. atisine, beiwutine, deltaline, mesaconitine, (−) salsolinol, hypaconitine, aconitine and songoramine, are toxic. The processed drug contained the components anthranoyllcoctonine, neojiangyouaconitine, songorine, delgrandine, lipo-14-O-anisoylbikhaconine and the unknown components U1 and U5. There was a remarkable 10–12 fold decrease in the content of aconitine; mesaconitine and hypaconitine, with the processed drug containing 1.900, 0.962, and 0.119 mg/kg of the respective markers (see Table 4).

In case of *A*. *carmichaelii*, the unprocessed drug LC-MS profile (Figure 5A) shows fewer components before processing. Upon processing, the aqueous filtrate shows the presence of the components benzoylmesaconine, benzoylaconine, beiwutine, mesaconitine, aconitine, carmichaelline, atisine and the unknown components U1 and U6 (as indicated in Additional file 4: Figure S3-B). The processed drug profile (Figure 5D), shows the presence of carmichaelline, beiwutine and non-toxic components lipo-14-O-anisoylbikhaconine, dihydroatisine, senbusine B, senbusine C and the unknown components named U1 and U6. There was a 15–16 fold decrease in the toxic alkaloidal content of the unprocessed drug, with the values for aconitine, mesaconitine and hypaconitine obtained as 1.150, 1.729 and 2.829 mg/kg, respectively.

Like the other two drugs, the unprocessed drug LC-MS profile of *Aconitum kusnezoffii* (Figure 6A), has very few components as compared to the processed drug and the aqueous filtrate obtained from decoction. This suggests an active hydrolytic conversion of the DDA’s to MDA’s in the detoxification process. The aqueous filtrate (see Additional file 3: Figure S3-C of additional data file) was found to contain atisine, benzoylaconine, aconitine, mesaconitine and hypaconitine, which belong to the more toxic class of aconitine alkaloids. The processed drug, along with reduced content of benzoylaconine, benzoylmesaconine and (−) salsolinol, showed the presence of 14-O-veratoylneoline, hestisine, and lipo-14-O-anisoylbikhaconine (see Figure 6D). The aconitine, mesaconitine and hypaconitine contents were found to decrease to 2.050, 2.448 and 1.067 mg/kg respectively indicating an overall 7- fold decrease in the toxic alkaloid content.

After an extensive literature survey of constituents reported in Aconitum species and after exclusion of components found in the blank controls of solvents used for processing, we found 5 unknown compounds whose identity was not confirmed. The molecular features of m/z value, molecular formula and UV max absorption were obtained by UHPLC-Q-TOF/MS analysis. The unknown compound U1 has retention time (t_R_) 5.32 mins and m/z value 453.273. The molecular formula was found to be C_24_H_39_NO_7_. Compound U2, was found to exhibit a retention time (t_R_) 7.30 mins and m/z value 480.295. The molecular formula was found to be C_26_H_41_NO_7._ Compound U3 was found to have m/z value of 450.228 with retention time (t_R_) 9.10 and with molecular formula as C_27_H_31_NO_5_. The unknown compound U4 had a retention time (t_R_) of 17.60 mins and m/z value of 628.349. The molecular formula for U4 was found to be C_30_H_57_N_15_. Compound U5 was found to have molecular formula of C_32_H_45_NO_10_ with retention time (t_R_) 12.100 mins and m/z value 626.294. The compounds U4 and U5 have molecular weights closer to the reported diester and monoester alkaloids. Thus we infer that they may belong to the monoester group of alkaloids formed after hydrolysis, caused due to processing. Compound U6 was found to have a high molecular m/z value of 850.570 with the molecular structure as C_47_H_73_NO_11_. This indicates that, U6 may possibly belong to the high molecular weight lipoid group of alkaloids, which is further substantiated by its delayed retention time (t_R_) of 26.50 mins. for elution. Due to lack of sufficient literature and reference compounds, the exact identity of the unknown compounds could not be established. Nevertheless, the molecular characteristics estimated from UHPLC-Q-TOF/MS analyses are shown in Additional file 4: Table S1 (additional data).

There are several animal based studies which report the lethal toxicity of unprocessed drug in comparison to the processed drug in which the toxicity is drastically reduced. Also several quantitative analysis methods report the estimation of increase in the hydrolysed products like aconine, hypaconine and mesaconine in the processed form of drug [53-60]. Hson-Mou and Paul have compiled several studies for toxicity of many drugs that include aconitum [61]. It states that in mice, when the toxicities (LD_50_) of the processed pieces of Aconite by oral and intravenous injection were tested the values were found to be 17.42 and 3.516 g/kg respectively. And overall, the toxicity level of the processed drug in comparison to the unprocessed raw drug was between 1/350 and 1/5 times. For oral dosage the LD_50_ was higher than 100 g/kg. Furthermore, there are no studies that report the comparative detoxification studies of aconite by TCM and Ayurveda method and the above discussed reports further support the findings of our study.

## Experimental

### Collection of plant material

The dried unprocessed roots of *A*. *heterophyllum* were obtained from commercial Ayurvedic stores from Mumbai, India and the unprocessed roots of *A*. *carmichaelii*, and *A*. *kusnezoffii* were purchased from commercial herbal stores in China. The authentication of all the plant materials used for the study was carried out by Professor ZhongZhen Zhao and the voucher specimens were deposited in the Chinese Medicines Centre of Hong Kong Baptist University. Roots with varying sizes were collectively used for various processing methods.

### Chemicals and reagents

Methanol and acetonitrile (HPLC grade) used as solvents for the mobile phase were obtained from E. Merck (Darmstadt, Germany). Formic acid (HPLC grade - purity 96.0%) used as a modifier was purchased from Tedia Company Inc (U.S.A.). Ultra-pure water used in various procedures of the experiment was obtained from a Milli-Q water purification system (Millipore, Bedford, MA, U.S.A.). Standard aconitine, mesaconitine, and hypaconitine were obtained from Tauto Biotech Co. Ltd. Sanghai, China.

### Detoxification processes of samples

All the three selected species of aconite roots were subjected to the traditional *Shodhana* and TCM methods of processing. The aim of the present work was to study the effect of traditional processing methods on the reduction of toxic contents of aconite. The factors like particle sizes of the drug material, processing times and shape of the roots, significantly affect the extraction of analytes. To specifically depict the traditional detoxification process and derive precise conclusions about the changes occurring in constituents due to such processing, we have used all the above mentioned parameters for the process as mentioned in the traditional records, and none of them were modified [7,25]. After the detoxification process all the dried processed forms of the drug were powdered (mesh size 2–8), subjected to extraction with chloroform and diluted with methanol prior to analysis.

Also, the processing practice by common people or traditional medicine practitioners would involve random selection of roots of varying shapes. This is a crucial aspect for a drug as aconitum where the toxicity varies not only based upon the size but also the shape of the roots. For instance, in the ancient TCM record “*Origins of the Materia Medica”* (Ben Cao Yuan Shi, published in 1612) it is mentioned that the roots of *A*. *carmichaelii* that have more number of projections and are large in size will be more potent as compared to those which have fewer projections [62]. We have found similar results in one of our unpublished works, where we have compared the toxic components of Aconitum roots of various sizes and shapes. Thus, to avoid any bias occurring due to particular shape, we have carried out random sampling of the roots for this study.

For *Shodhana* treatment with cow urine, the coarse powdered drug (mesh size 2–8) was kept immersed in cow urine in a tray and exposed to direct sunlight for 3 days. Every 5.0 g of sample was immersed in about 60.0 mL cow urine. For *Shodhana* with cow milk, 5.0 g of each of the drugs were hung in bags made of muslin cloth and placed in baths of boiling milk for 5 hours.

For the TCM method recorded in the Chinese pharmacopoeia, the aconitum roots were macerated in water for about 18 hours. After maceration, few roots were representatively cut to check that there was no hard core inside. The roots were soaked until there was no hard core observed inside after cutting [7]. The roots were then boiled in fresh water until there was no white core observed in the larger size roots. This process required approximately 6 hours in our experiments. The roots were then allowed to air dry, cut into slices and dried.

### Preparation of sample solutions

The processed drugs were powdered to uniform particle size of (mesh size 2–8) for further extraction and analysis. About 0.5 g sample powder was accurately weighed and transferred into a 10-mL centrifuge tubes. About 5.0 mL of chloroform was added and the mixture was sonicated for 30 min, followed by centrifugation at 3000 rpm for 10 min. The obtained chloroform extracts were filtered through 0.45 μm filters and diluted further with methanol to obtain appropriate concentrations for analysis.

### Preparation of standard solutions

Stock solutions of aconitine, mesaconitine and hypaconitine were prepared individually in methanol (HPLC grade). Working solutions of standard compounds were prepared by appropriate dilutions of respective stock solutions to obtain concentration of 400 ng mL^-1^ each. Serial dilutions of mixed standard solutions of aconitine, mesaconitine and hypaconitine were prepared in the range 2–100 ng mL^-1^ for calibration curve estimation.

### Quantitative analysis by UHPLC-Q-TOF-MS

Ultra-performance liquid chromatography was carried out using an Agilent 6540 accurate – mass Q-TOF LC/MS (Agilent Technologies, U.S.A.) [63,64]. Separation of components in the samples was performed at 20°C, using a UPLC C_18_ analytical column (I.D. 1.7 μm, ACQUITY UPLC® BEH, dimensions: 2.1 mm × 100 mm, Waters, U.S.A.), attached with a C_18_ pre-column (2.1 mm × 5 mm, I.D. 1.7 μm, VanGuard TM BEH, Waters, U.S.A.).

The mobile phase consisted of a mixture of water (A) and acetonitrile (B), both containing 0.1% formic acid. The optimized linear gradient elution was as follows: 0–10 min, 5–25% B; 10–25 min, 25–75% B; 25–28 min, 75–100% B; 28–31 min, 100–100% B; 31–31.1 min, 100–5% B with 2.9 min of equilibrium time. The injection volume used for analysis was 2 μL. The flow rate was kept constant at 0.4 mL/min. The acquisition of mass spectra was done in positive mode by scanning from 110 to 1700 in mass to charge ratio (*m/z*). The operation parameters for MS analysis were as follows: dry gas (N_2_) flow rate 6 L/min, nebulizer pressure 40 psi, dry gas temperature 300°C, Vcap 4500, fragmentor voltage 150 V and nozzle voltage 500 V.

The contents of aconitine, mesaconitine and hypaconitine were estimated in the processed and unprocessed samples. The average values of triplicate estimations of the contents of marker compounds were calculated and expressed as their amounts in processed and unprocessed samples.

### Validation of developed analytical method

The validation of the developed method was carried out in accordance with the ICH guidelines - Q2R1 (ICH, 2005). The parameters considered for validation were linearity, limit of detection (LOD) and limit of quantitation estimation (LOQ), accuracy (recovery studies), and precision (intraday, interday and repeatability) studies.

The linearity studies were carried out by 6 point calibration method with triplicate analysis at each selected concentration for all three standard compounds. Suitable concentrations were selected and analyzed in order to obtain a good correlation coefficient value (r^2^ ≥ 0.99).

The LOD and LOQ were estimated by analysis at several concentrations of analytes, diluted appropriately to estimate the lowest possible concentration that can be detected and quantitated with the optimized experimental conditions. The standard deviations of the responses and the slopes of the calibration curves were used to obtain the value for LOD and LOQ of each of the marker compounds.

Precision of the method was analyzed by three parameters, *viz*. interday-precision, intraday precision and repeatability studies. Repeatability studies were carried out by determinations of analytes in five injections of samples. Intraday precision was carried out by determination of analytes concentration in sample injections on the same day injected at five time intervals, namely, 0, 2, 4, 8 and 12 hrs. Interday precision was performed by determination of analytes in samples once a day for 3 consecutive days. The% RSD for all the determinations included in precision studies were calculated.

The accuracy of the developed method was estimated by performing recovery studies at three different concentration levels in triplicate within the linear range of the analytes. The recovery of the known added amount of markers added to the samples with known concentrations of markers were calculated and expressed as percentages of recovery.

### Data analysis

The analysis of data was carried out with Agilent Mass Hunter Workstation software-Qualitative Analysis (version B 4.00, Build 4.0.479.5, Service Pack 3, Agilent Technologies, Inc. 2011). The parameters adopted for analysis were: peaks with height ≥ 2000 counts; extraction restricted retention time 1.0-25.0 min, charge state considered was 1; peak spacing tolerance of 0.0025 m/z, plus 7.0 ppm; compound relative height ≥ 2.5%, and absolute height ≥ 1500 counts; for elements of C, H, O, N from 3–60, 0–120, 0–30, 0–30 respectively for generating formulae. Results were indicated with the help of base peak chromatograms (BPC with m/z range 150–950) for each sample analysed. The statistical data analysis was performed by Graph Pad INSTAT software (version 3.01).

## Conclusions

In the present study, three detoxification strategies comprising of *Shodhana* treatment with cow milk and cow urine and TCM process of aqueous decoction were compared. The order of efficiencies of the three processes for detoxification can be stated as: Processing with water > *Shodhana* with cow milk > *Shodhana* with cow urine. From the study we can infer that all the three methods of detoxification compared in this study are efficient in detoxification. This is the first study to report comparative study on the traditional experiences of processing of Aconite roots between Ayurveda and TCM. Also, to the best of our knowledge, this is the first study to explore the chemical profile of aconitum roots after treating by two forms of *Shodhana* (an ancient Ayurveda detoxification process) and for quantitation of the toxic alkaloids before and after processing.

We have applied these ancient methods of detoxification from traditional systems of two different countries and substantiated their validity through comparative experimental evidence. The developed analysis method finds its distinction in being the first study to demonstrate the comparative quantitative analysis and method validation of *Shodhana* treated aconitum by using the advanced and sensitive technique UHPLC-Q-TOF-MS. An easy and effective detoxification process by use of readily available solvents like water, cow urine and cow milk is suggested, and the process can be carried out easily by the consumers themselves. The present study provides better understanding of the processing of herbal drugs and can contribute towards the convergence of the ancient wisdom from different geographical and scientific backgrounds, thus supporting the concept of globalization of herbal medicine.

## Competing interests

The authors declare that they have no competing interests.

## Authors’ contributions

YJ has carried out the experiments for the study, collected the data and analysed them and has written the manuscript. ZZ and ZL have been significantly involved by contributing their intellectual content for the research work, analyzing the results and correcting the manuscript accordingly. HC and PY have made their intellectual contributions in revising the manuscripts with their knowledgeable suggestions. The final manuscript has been read and approved by all the authors.

## Supplementary Material

Additional file 1: Figure S1 Representative LC-MS base peak chromatograms of milk filtrate after Shodhana treatment of aconite roots (A) *A. heterophyllum* (B) *A*. *carmichaelii* (C) *A*. *kusnezoffii* (D) Cow milk filtrate used as control. Abbreviations used: S-(-) Salsolinol, AT- Atisine, BA- Benzoylaconine, BMA-Benzoylmesaconine, SG- Songoramine, BW- Beiwutine, M-Mesaconitine, BW- Beiwutine , C- carmichaelline, LP- Lipo-14-O-anisoylbikhaconine, SBC- Senbusine-C, A- Aconitine, H- Hypaconitine. Click here for file

Additional file 2: Figure S2 Representative LC-MS base peak chromatograms of cow urine filtrate after Shodhana treatment of aconite roots (A) *A. heterophyllum* (B) *A*. *carmichaelii* (C) *A*. *kusnezoffii* (D) Cow urine used as control. Abbreviations used: C- carmichaelline, AT- Atisine, BA- Benzoylaconine, BW- Beiwutine, M-Mesaconitine, A- Aconitine, SB- Senbusine-C, SG- Songoramine, H- Hypaconitine. Click here for file

Additional file 3: Figure S3 Representative LC-MS base peak chromatograms of aqueous filtrate after TCM treatment of aconite roots (A) *A. heterophyllum* (B) *A*. *carmichaelii* (C) *A*. *kusnezoffii* (D) Water used as control. Abbreviations used: S-(-) Salsolinol, AT- Atisine, BA- Benzoylaconine, BW- Beiwutine, M-Mesaconitine, A- Aconitine, H-Hypaconitine, BMA- Benzoylmesaconine, LP- Lipo-14-O-anisoylbikhaconine, SB- Senbusine – B. Click here for file

Additional file 4: Table S1 Data for peaks of various constituents identified in the three selected Aconitum species. Footnote: Key: U1-U6 are the unknown constituents detected in various samples of selected Aconitum species. Click here for file

Additional file 5: Table S2 List of molecular features of constituents present in the filtrate of cow milk and cow urine used for extraction of aconitum samples. Click here for file

Additional file 6: Figure S5 Representation of the mechanism of formation of MDA compounds from DDA components of Aconitum alkaloids by successive loss of chemical groups and respective changes in *m/z* values. Click here for file

Additional file 7: Figure S4 Representative LC-MS-ESI scan spectra of various filtrates obtained after processing *A. carmichaelii* for depicting conversion of DDA compounds to MDA. (A) Unprocessed drug (B) Filtrate obtained after extraction with Cow urine (C) Filtrate obtained after extraction with Cow milk (D) Filtrate obtained after extraction with water. The MDA components and hydrolyzed products formed from DDA compounds are indicated with a red asterisk (*) symbol. Click here for file
