# Supplementary figures and images for: A comparative study on the traditional Indian Shodhana and Chinese processing methods for aconite roots by characterization and determination of the major components
Source: Chem Cent J. 2013 Oct 25;7:169. doi: 10.1186/1752-153X-7-169 (PMC4015782; doi:10.1186/1752-153X-7-169)

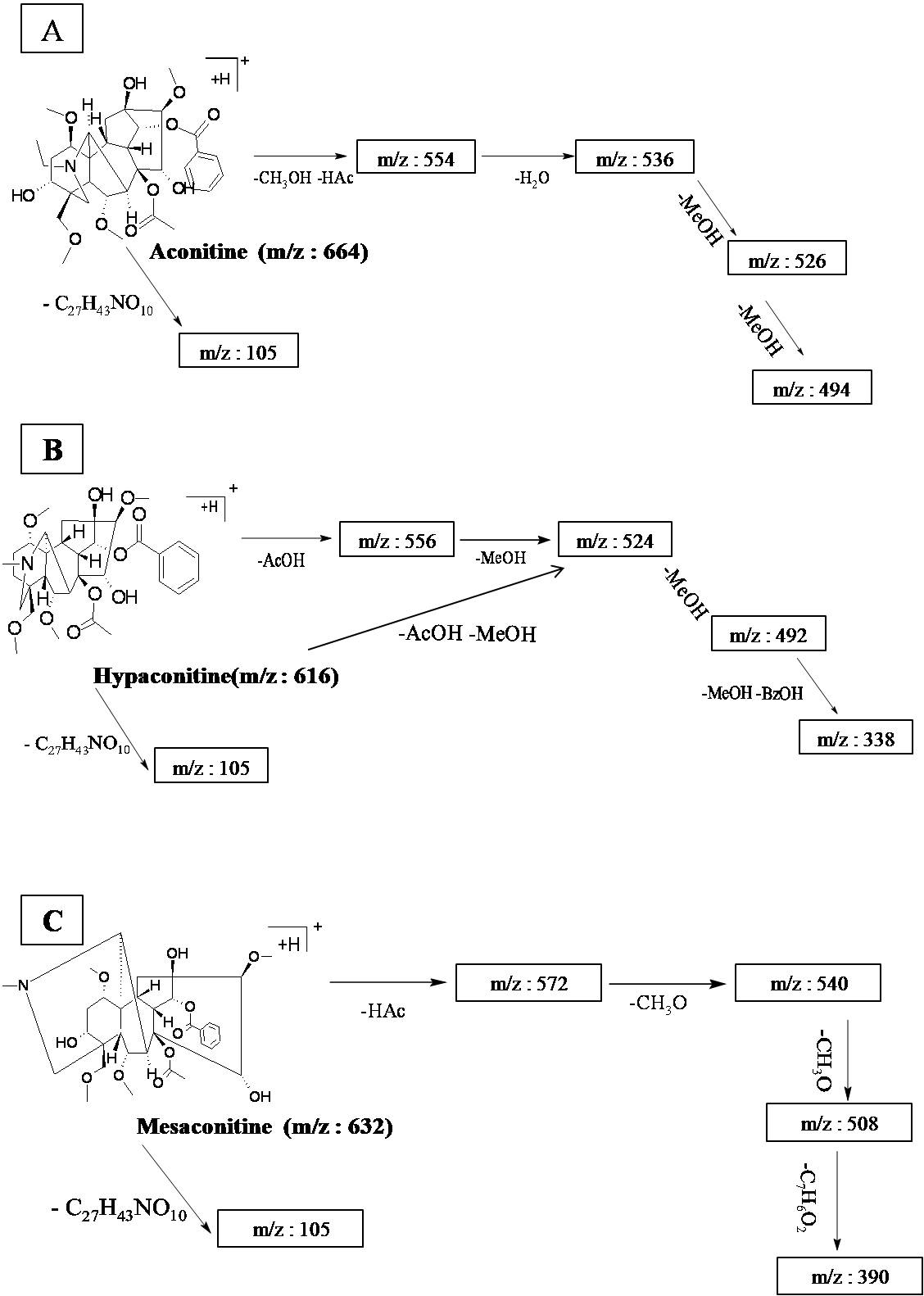

Supplement: Additional file 1: Figure S1 — Representative LC-MS base peak chromatograms of milk filtrate after Shodhana treatment of aconite roots (A) A. heterophyllum (B) A. carmichaelii (C) A. kusnezoffii (D) Cow milk filtrate used as control. Abbreviations used: S-(-) Salsolinol, AT- Atisine, BA- Benzoylaconine, BMA-Benzoylmesaconine, SG- Songoramine, BW- Beiwutine, M-Mesaconitine, BW- Beiwutine , C- carmichaelline, LP- Lipo-14-O-anisoylbikhaconine, SBC- Senbusine-C, A- Aconitine, H- Hypaconitine. [file 1752-153X-7-169-S1.jpeg]

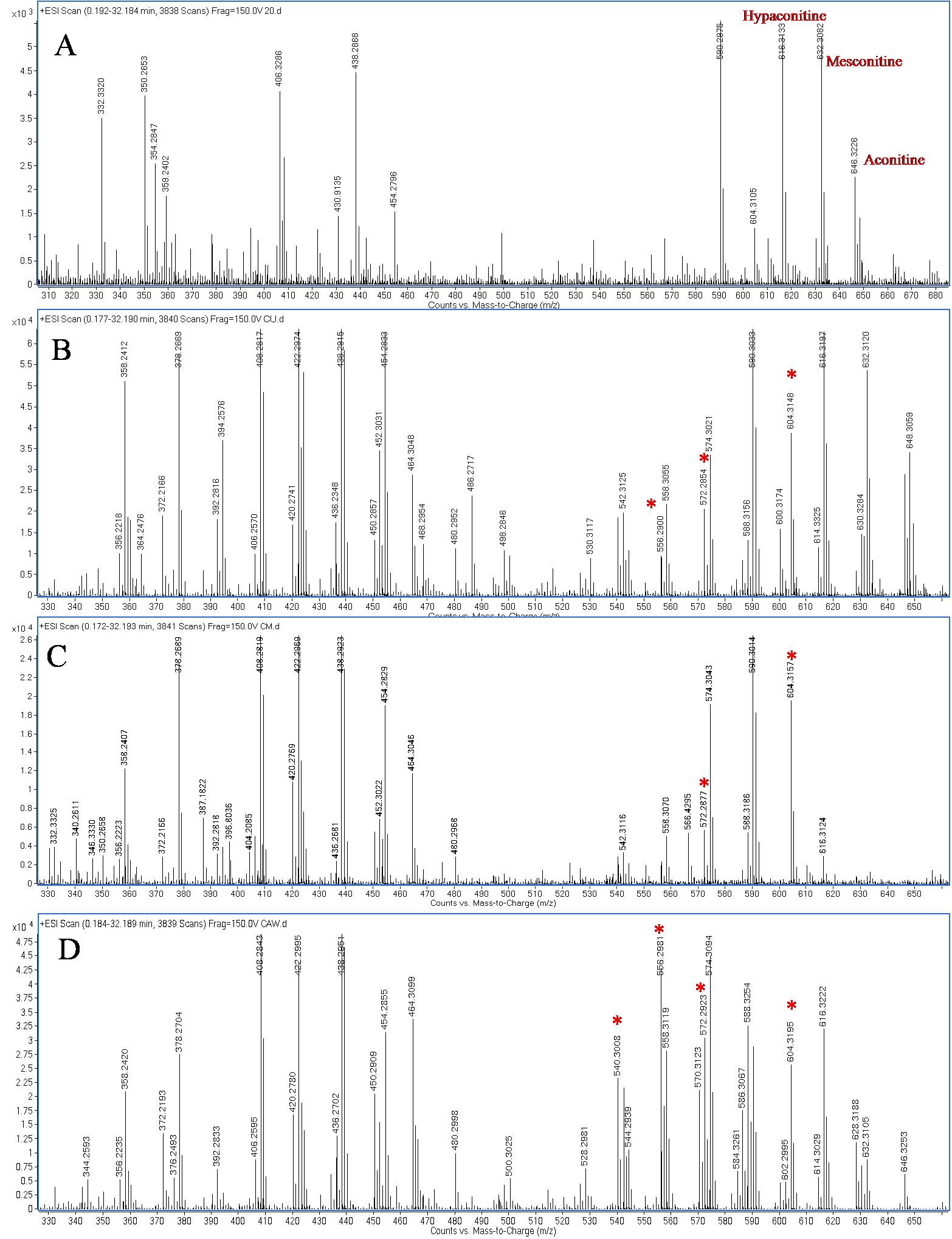

Supplement: Additional file 2: Figure S2 — Representative LC-MS base peak chromatograms of cow urine filtrate after Shodhana treatment of aconite roots (A) A. heterophyllum (B) A. carmichaelii (C) A. kusnezoffii (D) Cow urine used as control. Abbreviations used: C- carmichaelline, AT- Atisine, BA- Benzoylaconine, BW- Beiwutine, M-Mesaconitine, A- Aconitine, SB- Senbusine-C, SG- Songoramine, H- Hypaconitine. [file 1752-153X-7-169-S2.jpeg]

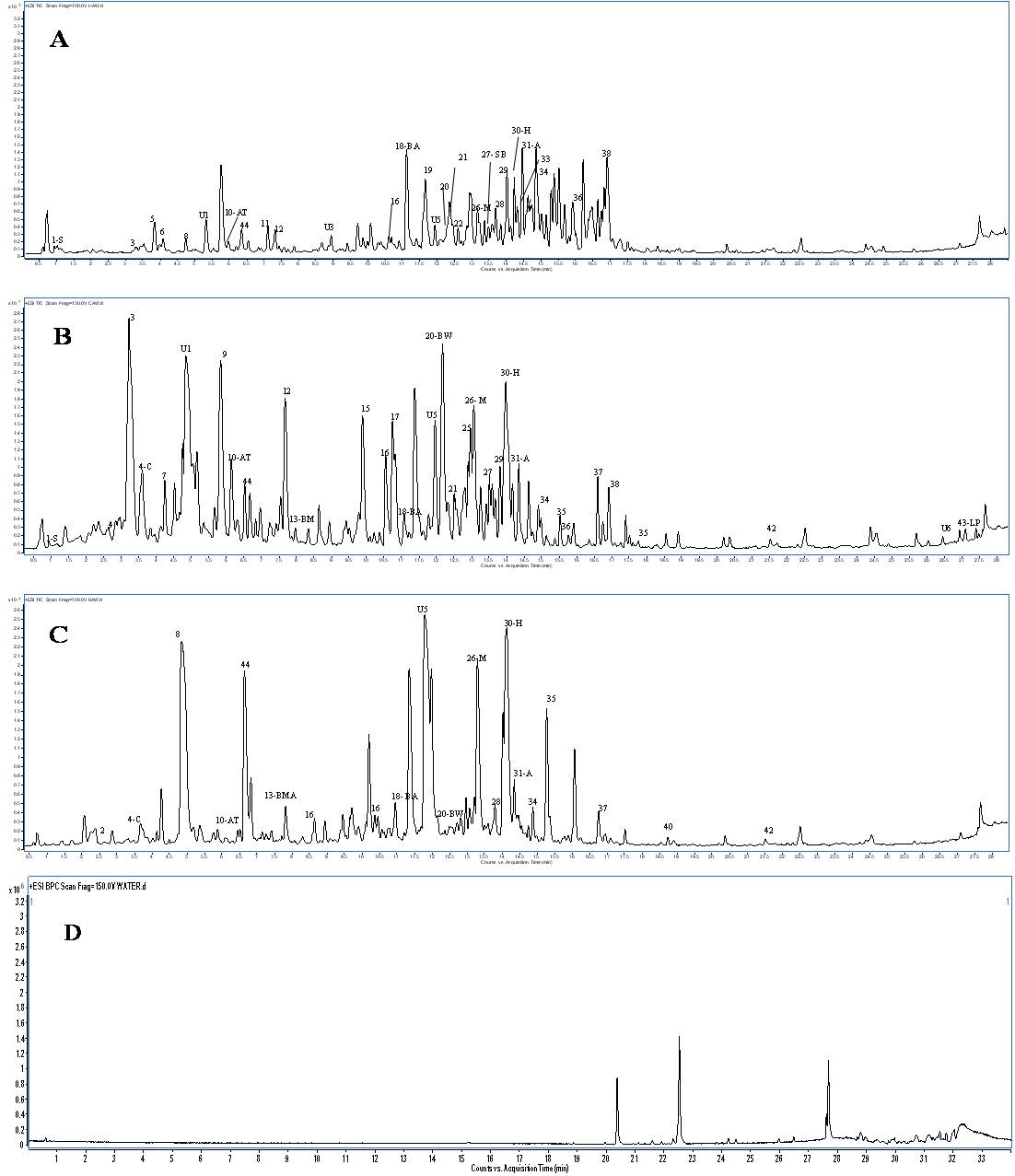

Supplement: Additional file 3: Figure S3 — Representative LC-MS base peak chromatograms of aqueous filtrate after TCM treatment of aconite roots (A) A. heterophyllum (B) A. carmichaelii (C) A. kusnezoffii (D) Water used as control. Abbreviations used: S-(-) Salsolinol, AT- Atisine, BA- Benzoylaconine, BW- Beiwutine, M-Mesaconitine, A- Aconitine, H-Hypaconitine, BMA- Benzoylmesaconine, LP- Lipo-14-O-anisoylbikhaconine, SB- Senbusine – B. [file 1752-153X-7-169-S3.jpeg]
