# Supplementary material for: A comparative study on the traditional Indian Shodhana and Chinese processing methods for aconite roots by characterization and determination of the major components
Source: Chem Cent J. 2013 Oct 25;7:169. doi: 10.1186/1752-153X-7-169 (PMC4015782; doi:10.1186/1752-153X-7-169)
Supplement: Additional file 4: Table S1 — Data for peaks of various constituents identified in the three selected Aconitum species. Footnote: Key: U1-U6 are the unknown constituents detected in various samples of selected Aconitum species. [file 1752-153X-7-169-S4.docx]

**Table T1** Data for peaks of various constituents identified in the three selected Aconitum species

| **No.** | | **Name of constituent** | **m/z values** | **Molecular formula** | **Retention time (min.)** |
| --- | --- | --- | --- | --- | --- |
|  | (-)-Salsolinol | | 180.1024 | C_10_H_13_NO_2_ | 1.140 |
|  | Hetisinone | | 328.1905 | C_20_H_25_NO_3_ | 2.640 |
|  | Karakanine | | 376.2443 | C_22_H_33_NO | 3.237 |
|  | Carmichaelline | | 378.2644 | C_22_H_35_NO_4_ | 3.555 |
|  | Songorine | | 358.2386 | C_22_H_31_NO_3_ | 3.713 |
|  | Hestisine | | 330.2068 | C_20_H_27_NO_3_ | 4.027 |
|  | Lepenine | | 360.2542 | C_22_H_33_NO_3_ | 4.100 |
|  | Delphatine | | 504.2949 | C_26_H_43_NO_7_ | 4.664 |
|  | Talatizamine | | 422.2907 | C_24_H_39_NO_5_ | 6.015 |
|  | Atisine | | 344.2619 | C_22_H_33_NO_2_ | 6.071 |
|  | Chasmanine | | 452.3002 | C_25_H_41_NO_6_ | 7.017 |
|  | Dihydroatisine | | 346.2743 | C_22_H_35_NO_2_ | 7.593 |
|  | Benzoylmesaconine | | 606.2903 | C_31_H_43_NO_11_ | 8.095 |
|  | Songoramine | | 356.2227 | C_22_H_29_NO_3_ | 8.191 |
|  | Lappaconitine | | 584.3097 | C_32_H_44_N_2_O_8_ | 9.930 |
|  | Anthranoyllcoctonine | | 609.3131 | C_32_H_46_N_2_O_8_ | 10.578 |
|  | Hokbusine b | | 476.2428 | C_27_H_35_NO_5_ | 10.993 |
|  | Benzoylaconine | | 604.3112 | C_32_H_45_NO_10_ | 11.001 |
|  | Delbrusine | | 516.2952 | C_27_H_43_NO_7_ | 11.849 |
|  | Beiwutine | | 648.3008 | C_33_H_45_NO_12_ | 12.418 |
|  | Delbruline | | 466.2867 | C_25_H_39_NO_7_ | 12.563 |
|  | Lycoctonine | | 468.2912 | C_25_H_41_NO_7_ | 12.705 |
|  | Neojiangyouaconitine | | 602.3323 | C_33_H_47_NO_9_ | 12.765 |
|  | Deltaline | | 530.2746 | C_27_H_41_NO_8_ | 12.852 |
|  | 14-o-veratoylneoline | | 588.3245 | C_33_H_47_O_9_ | 13.367 |
|  | Mesaconitine | | 632.3058 | C_33_H_45_NO_11_ | 13.368 |
|  | Senbusine C | | 907.5621 | C_24_H_39_NO_7_ | 13.525 |
|  | Aconifine | | 662.3162 | C_34_H_47_NO_12_ | 13.879 |
|  | Ignavine | | 450.2276 | C_27_H_31_NO_5_ | 14.268 |
|  | Hypaconitine | | 616.3151 | C_33_H_45_NO_10_ | 14.271 |
|  | Aconitine | | 646.3211 | C_34_H_47_NO_11_ | 14.328 |
|  | Isodelphinine | | 600.3162 | C_33_H_45_NO_9_ | 14.444 |
|  | 14-O-Anisoylneoline | | 572.3210 | C_32_H_45_NO_8_ | 14.455 |
|  | Yunaaconitine | | 660.3392 | C_35_H_49_NO_11_ | 15.020 |
|  | Delgrandine | | 742.2858 | C_41_H_43_NO_12_ | 15.618 |
|  | 3-deoxyaconitine | | 630.3265 | C_34_H_47_NO_10_ | 15.627 |
|  | 8-O-Cinnamoylneoline | | 364.1970 | C_23_H_25_NO_3_ | 16.698 |
|  | Vilmorrianine C | | 628.3472 | C_35_H_49_NO_9_ | 17.059 |
|  | Senbusine B | | 446.2535 | C_23_H_37_NO_6_ | 18.678 |
|  | Bullatine B | | 437.5695 | C_24_H_39_NO_6_ | 18.681 |
|  | Heteratisine | | 783.4706 | C_22_H_33_NO_5_ | 19.473 |
|  | Demethyldelavaine A | | 701.3694 | C_37_H_52_N_2_O_11_ | 21.656 |
|  | Lipo-14-O-anisoylbikhaconine | | 840.5646 | C_49_H_77_NO_10_ | 27.409 |
|  | 8-deoxy-14-dehydro aconosine | | 359.2459 | C_22_H_33_NO_3_ | 6.510 |
|  | | | | | |
| **List of peaks for unknown constituents** | | | | | |
|  | | | | | |
| **UV λ _max_**  **(nm)** | | | **m/z values** | **Molecular formula** | **Retention time (mins.)** |
| U1 | 220 | | 437.569 | C_24_H_39_NO_6_ | 5.325 |
| U2 | 230 | | 480.295 | C_26_H_41_NO_7_ | 7.302 |
| U3 | 220 | | 450.228 | C_27_H_31_NO_5_ | 9.100 |
| U4 | 222 | | 628.349 | C_30_H_57_N_15_ | 17.600 |
| U5 | 221 | | 626.294 | C_32_H_45_NO_10_ | 12.100 |
| U6 | 224 | | 850.570 | C_47_H_73_NO_11_ | 26.500 |

**Key:** U1-U6 are the unknown constituents detected in various samples of selected Aconitum species
