# Supplementary material for: A comparative study on the traditional Indian Shodhana and Chinese processing methods for aconite roots by characterization and determination of the major components
Source: Chem Cent J. 2013 Oct 25;7:169. doi: 10.1186/1752-153X-7-169 (PMC4015782; doi:10.1186/1752-153X-7-169)
Supplement: Additional file 5: Table S2 — List of molecular features of constituents present in the filtrate of cow milk and cow urine used for extraction of aconitum samples. [file 1752-153X-7-169-S5.docx]

**Additional data file 5:**

**Table no. T2** List of molecular features of constituents present in the filtrate of cow milk and cow urine used for extraction of aconitum samples

| **S. No** | **Retention time** | **m/z value** | **Mass** | **Molecular formula** | **Δ ppm** |
| --- | --- | --- | --- | --- | --- |
| **Components present in Cow milk filtrate** | | | | | |
|  | 4.608 | 340.263 | 339.253 | C_18_ H_33_N_3_O_3_ | -4.32 |
|  | 6.300 | 436.270 | 435.263 | C_25_H_33_N_5_O_2_ | 0.14 |
|  | 6.456 | 453.347 | 452.339 | C_29_H_44_N_2_O_2_ | 1.55 |
|  | 7.850 | 566.431 | 565.424 | C_35_H_55_N_3_O_3_ | 0.46 |
|  | 8.922 | 679.515 | 678.508 | C_41_H_66_N_4_O_4_ | 0.11 |
|  | 9.836 | 218.214 | 217.206 | C_12_H_27_NO_2_ | -2.31 |
|  | 10.453 | 234.207 | 233.200 | C_12_H_27_NO_3_ | -1.2 |
|  | 12.100 | 524.302 | 523.294 | C_32_H_37_N_5_O_2_ | 0.43 |
|  | 14.328 | 290.271 | 289.264 | C_16_H_35_NO_3_ | -2.57 |
|  | 17.024 | 274.274 | 273.267 | C_16_H_35_NO_2_ | -0.68 |
|  | 18.494 | 415.212 | 414.205 | C_25_H_26_N_4_O_2_ | 0.59 |
|  | 19.320 | 379.154 | 378.144 | C_23_H_22_O_5_ | 4.79 |
|  | 19.392 | 302.308 | 301.301 | C_18_H_39_NO_2_ | -2.87 |
|  | 20.301 | 256.300 | 255.293 | C_17_ H_37_N | -0.38 |
|  | 21.110 | 296.258 | 295.252 | C_18_H_33_NO_2_ | -2.15 |
|  | 21.526 | 374.363 | 373.356 | C_22_H_47_NO_3_ | -0.63 |
|  | 22.089 | 400.378 | 399.372 | C_24_H_49_NO_3_ | -1.76 |
|  | 22.750 | 522.250 | 521.242 | C_31_H_31_N_5_O_3_ | 1.0 |
|  | 22.761 | 332.334 | 331.326 | C_23_H_41_N | -2.86 |
|  | 27.831 | 584.561 | 583.554 | C_36_H_73_NO_4_ | -0.25 |
|  | 30.983 | 647.460 | 646.453 | C_39_H_18_O2 | -1.01 |
|  | 31.734 | 537.395 | 536.388 | C_36_H_48_N_4_ | -0.46 |
| **Components present in Cow urine filtrate** | | | | | |
|  |  |  |  |  |  |
|  | 1.730 | 394.260 | 393.251 | C_23_H_31_N_5_O | 2.7 |
|  | 2.430 | 424.269 | 423.262 | C_23_H_37_NO_6_ | -0.72 |
|  | 4.243 | 438.285 | 437.277 | C_24_H_39_NO_6_ | 0.44 |
|  | 4.523 | 340.263 | 339.254 | C_18_H_33_N_3_O_3_ | -1.78 |
|  | 4.882 | 438.286 | 437.278 | C_25_H_35_N_5_O_2_ | 0.16 |
|  | 5.089 | 420.274 | 419.267 | C_24_H_37_NO_5_ | -0.28 |
|  | 5.195 | 468.295 | 467.288 | C_25_H_41_NO_7_ | 0.36 |
|  | 5.730 | 392.279 | 391.272 | C_23_H_37_NO_4_ | 0.45 |
|  | 6.086 | 342.242 | 341.235 | C_22_H_31_NO_2_ | -0.22 |
|  | 6.524 | 452.300 | 451.293 | C_25_H_41_NO_6_ | 0.59 |
|  | 6.391 | 453.347 | 452.3399 | C_29_H_44_N_2_O_2_ | 0.38 |
|  | 7.701 | 464.302 | 463.295 | C_27_H_37_N_5_O_2_ | -0.21 |
|  | 7.910 | 606.290 | 605.283 | C_31_H_43_NO_11_ | 0.27 |
|  | 8.610 | 498.285 | 497.277 | C_29_H_39_NO_6_ | 0.99 |
|  | 9.160 | 701.499 | 700.491 | C_42_H_68_O_8_ | 0.34 |
|  | 9.430 | 574.301 | 573.293 | C_31_H_43_NO_9_ | 0.71 |
|  | 9.619 | 514.279 | 513.272 | C_29_H_39_NO_7_ | 0.19 |
|  | 10.102 | 209.154 | 208.147 | C_13_H_20_O_2_ | -0.65 |
|  | 10.169 | 590.297 | 589.291 | C_32_H_39_N_5_O_6_ | -3.18 |
|  | 11.500 | 574.301 | 573.294 | C_31_H_43_NO_9_ | -0.31 |
|  | 11.553 | 209.132 | 208.124 | C_16_H_16_ | 0.31 |
|  | 13.128 | 646.322 | 645.314 | C_34_H_47_NO_11_ | 0.88 |
|  | 14.196 | 246.245 | 245.238 | C_14_H_31_NO_2_ | -2.49 |
|  | 14.732 | 614.332 | 613.324 | C_34_H_47_NO_9_ | 0.53 |
|  | 17.012 | 319.307 | 318.299 | C_16_H_38_N_4_O_2_ | -0.17 |
|  | 19.447 | 318.302 | 317.295 | C_18_H_39_NO_3_ | -2.3 |
|  | 20.530 | 316.321 | 315.313 | C_19_H_41_NO_2_ | -0.26 |
|  | 21.392 | 322.270 | 321.264 | C_15_H_35_N_3_O_4_ | -1.85 |
|  | 21.659 | 330.340 | 329.332 | C_20_H_43_NO_2_ | -3.38 |
